# Simulators of Superconductor Critical Current: Design, Characteristics, and Applications

**DOI:** 10.6028/jres.096.046

**Published:** 1991

**Authors:** L. F. Goodrich, A. N. Srivastava, T. C. Stauffer

**Affiliations:** National Institute of Standards and Technology, Boulder, CO 80303

**Keywords:** critical current, data acquisition, data analysis, electronic circuit, high-*T*_c_, low-*T*_c_, measurement method, standard, superconductor, superconductor simulator, voltage-current characteristic

## Abstract

The superconductor simulator is an electronic circuit that emulates the extremely nonlinear voltage-current characteristic (the basis of a critical-current measurement) of a superconductor along with its other major electrical properties. Three different types of simulators have been constructed: the passive, active, and hybrid simulator. The passive simulator has the fewest circuit components and offers the least amount of versatility, while the active and hybrid simulators offer more versatility and consequently have more components. Design, characteristics, and applications of the superconductor simulator along with a summary of features are presented. These simulators are high precision instruments, and are thus useful for establishing the integrity of part of a superconductor measurement system. They are potentially useful for testing the measurement method and data acquisition and analysis routines. The 50 A simulator provides critical-current precision of 0.1% at a 1 μV signal. This is significantly higher than the precision of a superconducting standard reference material. The superconductor simulator could significantly benefit superconductor measurement applications that require high-precision quality assurance.

## 1. Introduction

The superconductor simulator emulates the extremely nonlinear voltage-current characteristics of a superconductor. By measuring this characteristic, a “critical current” of the simulator can be obtained. Thus, the simulator can aid in determining the integrity of part of a critical-current measurement system.

A superconductor’s critical current (*I*_c_) is a measure of its current carrying capacity, and is defined as that current at which a specified electric-field criterion (*E*_c_), or resistivity criterion (*ρ*_c_) is achieved in the specimen.

The voltage-current (*V-I*) characteristic of the superconductor can often be modeled by the empirical equation:

$$V=V_{o}{\left(I/I_{o}\right)}^{n}$$ (1)

where *I*_o_ is the observed current at a voltage *V*_o_, and *n* reflects the abruptness of the transition from the superconducting to the normal state. Typical values of *n* range from 10 to 100. For a more complete discussion of the definition of critical current and the *V-I* characteristic, consult Refs. [1,2]. The superconductor simulator’s critical current is determined in the same manner as for a superconductor.

The process of measuring the critical current of a superconductor is challenging, since it requires measuring low voltages under high current conditions. The critical-current standard-test-method of the American Society for Testing and Materials has an uncertainty of ±5%. The superconductor simulator’s precision is on the order of 0.1% with a 1 μV signal level. This level of precision exceeds the recommendations of the ASTM for present superconductor measurements.

The simulator provides a simple, expedient, and repeatable test of a complex measurement system. Another test method using a *I*_c_ standard reference material, SRM 1457 (low-*T*_c_, Cu/Nb-Ti wire) exists. However, it has enormous startup costs if the experimenter does not already have a high current and high magnetic field capability. This single-use device also requires the ability to operate at liquid helium temperatures. High-*T*_c_ reference materials do not exist yet, and could be subject to degradation and other instabilities. The simulator operates at room temperature and is a multiple-use device, since its characteristics are stable with time and use. It can also be used with both high-*T*_c_ and low-*T*_c_ measurement systems.

Three different types of simulators have been designed: the passive, hybrid, and the active simulator. The passive simulator consists of non-active, low-reactance components. Thus, its electrical characteristics should not significantly depend on frequency in the region of interest. This device is useful in comparing measurement methods with different frequency characteristics, such as the dc and ac methods or the dc and pulse methods. For a complete discussion of these measurement methods, see Refs. [3,4],

The passive simulator can be considered to be a sample substitution box; the experimenter connects the simulator to the measurement system as though it were a superconductor. Since the simulator operates at room temperature, its temperature dependence was extensively studied. Thus, voltage and current measurements and data analysis routines can be thoroughly tested. After a successful test is performed on the simulator, the integrity of part of the measurement system can be established.

The hybrid simulator has the same electrical components as the passive simulator, but it also contains an insulated oven with an electronic temperature controller. The temperature controller keeps the diode, a key component of the circuit, at a constant temperature. Thus, the response of the simulator will be less a function of room temperature.

The active simulator has operational amplifiers, a temperature controller for the diode, and other active components in its circuitry. These components may have some frequency dependence, since wire wound resistors were used and the gain of the op-amps may depend on frequency. Thus, this simulator could yield different critical currents depending upon which measurement method is used. It is therefore only intended to be used with the dc measurement method. The active components allow the user to select the values of *n* and *I_c_.*

Although the active simulator’s critical current may vary with measurement method, given a measurement technique, it can test the ability of a data acquisition and analysis routine to handle widely varying values of *n* and *I_c_.* Since data acquisition and analysis to determine *I*_c_ with a large variability in *n* can be difficult, the active simulator was designed to have a wide range of *n* values. The range of *n* for the active simulator is from 25 to 150. Values of *n* much greater than 150 are possible, but they are not relevant for superconductor characterization at this time.

The active simulator, unlike a passive or hybrid simulator, is not a sample substitution box. Instead of being connected to the measurement system as an actual sample, this simulator takes its input from the current supply’s current shunt, not the output current of the supply. Thus the *h* of one active simulator could be 10 mA to 10 kA depending on the resistive value of the current supply’s current shunt.

## 2. Simulator Operation and Design Considerations

The simulators described here rely on a diode (Zener diode #1N5252B) to generate a nonlinear voltage-current characteristic. It is important to realize that in a superconductor, the voltage increases from zero abruptly near a certain current. In a diode, however, the current increases from zero abruptly near a certain voltage. Thus, to emulate a superconductor, the diode characteristic must be “inverted” so that near a specified current the measured voltage increases.

Passive and hybrid simulators have a single, preset *n* characteristic and can have many different values of *I_c_.* The value of *n* varies slightly with voltage; the quiescent value of *n* is mainly determined by the diode, and is selectable by using diodes with different voltage-current characteristics. These simulators have an *n* -value of approximately 25, whereas the active simulator has a variable *n* -value feature. Passive and hybrid simulators are best suited for applications under 100 A, whereas the active simulator is suited for use over 100 A.

### 2.1 Passive and Hybrid Simulator Operation

**Figure 1:**
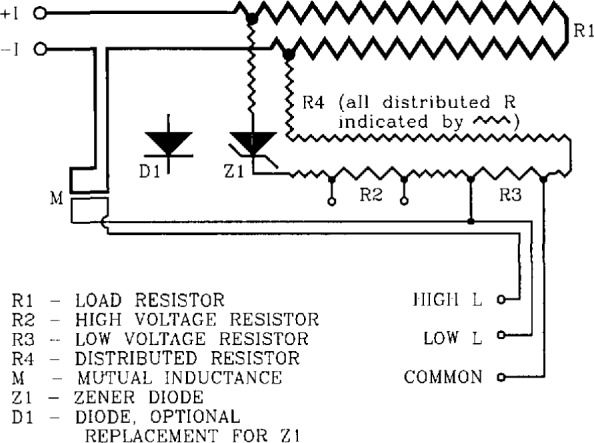
Circuit diagram for the passive simulator. The voltage signal across R2 is denoted as V2, and the signal across R3 is denoted as V3. The hybrid simulator has the same circuit, except the diode Z1 is enclosed in a temperature controller.

Figure 1 shows the circuit diagram for the passive simulator. The passive simulator consists of four resistors: R1, R2, R3, and R4, and a Zener diode Z1. The resistors are made from manganin resistance wire which has a low temperature dependence. A regular diode could be used in place of the Zener, although the values of *n* are generally lower for regular diodes. The resistor R1 is the main current branch; it carries most of the current from the power supply. The current branch connected in parallel with R1, called the parallel current branch, generates the desired voltage-current response.

The parallel current branch abruptly starts conducting current when the voltage drop across R1 is near the threshold voltage of the forward-biased Zener diode (approximately 0.7 V). The current through the parallel branch increases rapidly until the voltage across the current limiting resistors, R2 and R4 (R4 is a distributed resistance), becomes significant compared to 0.7 V. At this point, this resistance protects the diode from conducting too much current, which could lead to irreversible shifts in the *V-I* characteristic. The voltage drop across the series resistor R3 is the simulated sample voltage, and is denoted by V3. Since the value of resistor R2 is approximately 100 times greater than that of R3, the voltage signal on R2, V2, can be used to accurately infer the “actual” lower voltage signal V3. The voltage at R2 could be normalized by the measured ratio of resistances R3 and R2.

**Figure 2:**
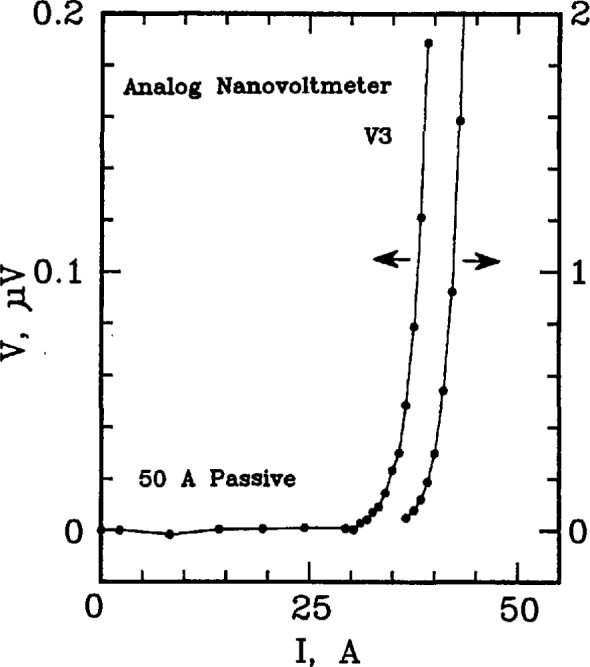
Linear plot of voltage versus current in the low voltage region of the SO A passive simulator; the voltage across R3 (V3) was measured with an analog nanovoltmeter. The right-most curve is a continuation of the data on an expanded scale, shown on the right-hand axis.

**Figure 3:**
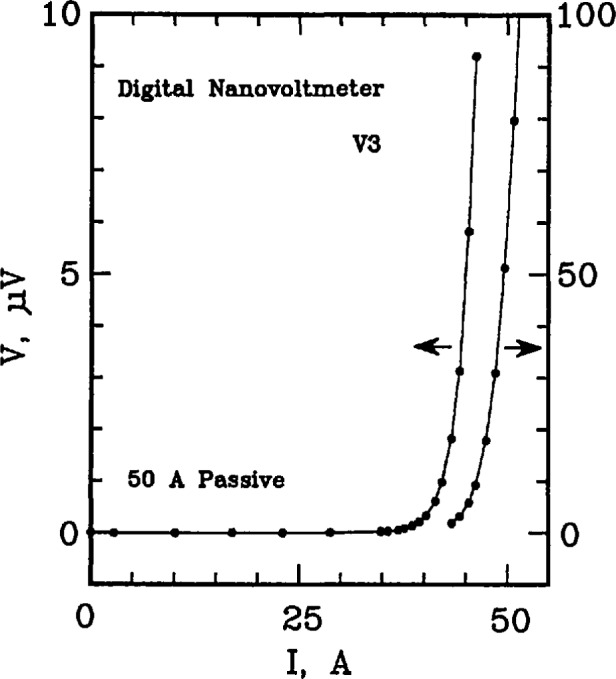
Linear plot of voltage versus current in the high voltage region of the SO A passive simulator; voltage across R3 (V3) was measured with a digital nanovoltmeter. The right-most curve is a continuation of the data on an expanded scale, shown on the right-hand axis.

Examples of *V-I* data from the 50 A passive simulator are given on Figs. 2 and 3. These curves were taken by measuring V3 on the simulator. An analog nanovoltmeter was used to obtain the voltage data on Fig. 2. The measured voltage at currents below 25 A was typically zero within ± 2 nV, which is at the noise level of the analog voltmeter. Measurements of V2 indicated that the actual voltage V3 was zero in this region to within ±0.1 nV. These results were typical for all simulators including the active simulator. Most of the characterization of the simulators was done at higher sample voltages with digital nanovoltmeters as shown on Fig. 3. The dynamic range of the digital voltmeters allows for characterization over a wide range of voltages. The range of interest is typically from 0.1 to 10 μV.

**Figure 4:**
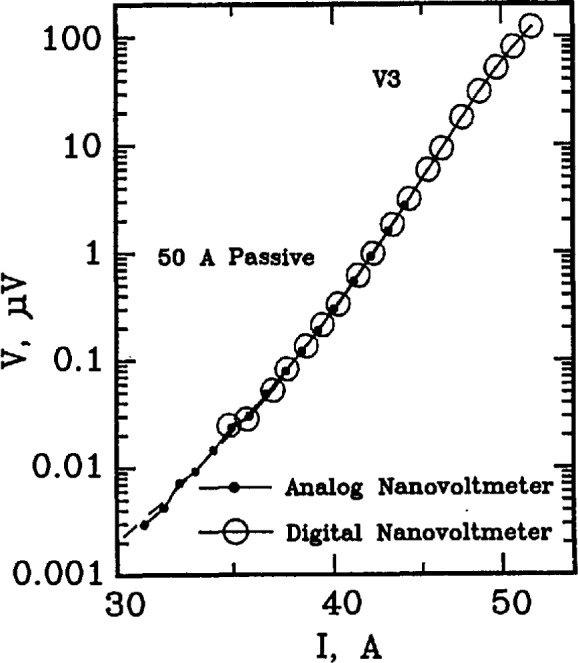
Full logarithmic plot of voltage versus current in the high- and low-voltage region of the SO A passive simulator.

To illustrate that the *V-I* characteristic of the simulator can be approximated by Eq. (1), a full logarithmic plot of the *V-I* characteristic is given on Fig. 4. This plot is linear, in accordance with the empirical equation:

$$\log\;V=\log\;V_{0}+n\cdot \log\left(I/I_{0}\right),$$ (2)

where *n* is the slope of this curve. The data on Fig. 4 are the same as those on Figs. 2 and 3. The dashed line in the low voltage region is the curve obtained by measuring the voltage across R2. The agreement is within a few nV on the low voltage region.

To obtain higher critical currents, the connections of the parallel branch can be moved to a smaller portion of the resistance wire R1. In effect, a voltage divider is created along R1, and a higher current will be needed to generate the 0.7 V diode threshold voltage. In this manner, the critical current is effectively increased. Likewise, moving the taps to a larger section of R1 decreases *I_c_.* Instead of physically moving the connections along R1, a low-thermal, low-resistance switch can be installed in the simulator to achieve the same effect.

The hybrid simulator is identical to the passive simulator except that it has a temperature-control circuit to maintain the diode Z1 at a nearly constant temperature. Thus, the temperature dependence of the hybrid simulator is significantly reduced.

### 2.2 Passive and Hybrid Simulator Design Considerations

When designing the passive simulator, care was taken to reduce unwanted effects of high self-inductance and power dissipation. The resistor R1 in Fig. 1 is made of manganin resistance wire (or a strip, for high current applications) and dissipates most of the power drawn from the current supply. It is therefore kept outside the simulator box, and away from the other circuit components to reduce thermoelectric voltages. In addition to physically separating R1 from the rest of the circuit, the parallel current branch was connected using manganin wire soldered to R1. These contacts also aided in reducing thermoelectric voltages. Regions of the circuit that are not subject to wide temperature variations use copper wire.

To further reduce thermoelectric effects, lead-shot bags (cloth bags containing lead-shot) were placed upon the R2-R3-Z1 circuitry, thus keeping each of these elements at nearly the same (but not necessarily constant) temperature. Thus, temperature gradients due to the heat from resistor R1 or the environment were reduced within the circuit. The voltage taps were constructed with continuous twisted 14 AWG copper wire to reduce thermoelectric effects. Also, as standard practice, the connections to the voltmeter inputs were thermally shielded using lead-shot bags.

Table 1 shows the resistance specifications for the active, hybrid, and passive simulators. The active simulator’s resistances will be discussed in Sec. 2.4. Resistors R1, R2, and R3 are defined as the resistance between their respective voltage taps. Resistor R4 is defined as the total distributed resistance in the parallel circuit branch. The 6th column of Table 1 is the ratio of R2 to R3, the significance of which will be discussed in detail later.

The values of R2, R3, and R4 were chosen to result in an *n* versus *V* characteristic that peaks in the region of 1 to 10 μV, which is the region of interest in superconductor characterization. The *n*-value is typically 25 for the passive and hybrid simulators. The small value of R3 was chosen to better approximate the impedance of a superconducting sample. However, an extremely small value of R3 would result in diode self-heating, since a larger current would be required to achieve the same signal level. The voltage tap separation on R2 was chosen such that the included resistance was about 100 times the resistance between the taps on R3, as indicated on Table 1.

To reduce the simulator’s temperature dependence, resistors R1, R2, R3, and R4 were made of manganin wire, whose resistance has a low temperature dependence. The parallel branch connection wires were also made of manganin (part of the distributed resistance R4). Thus, most of the temperature variation of the simulator could be attributed only to the diode. This dependence was on the order of 0.3% change in *I*_c_ per °C.

The resistor R1 is bifilar, meaning that it is wound back onto itself so that most magnetic flux lines cancel each other, thus reducing self inductance. High self inductances could lead to frequency dependent simulator characteristics. In this case, the simulator’s critical current would vary with measurement method.

In superconductors, a mutual inductance exists between the specimen and the specimen voltage taps due to the area enclosed between the specimen and the voltage tap leads. Although this inductance is unavoidable, it can be reduced by minimizing the area subtended by the voltage taps and the conductor. To emulate this mutual inductance, the main current branch and the voltage taps from the parallel current branch are brought into close proximity of one another. This inductive coupling cannot be achieved through the side of a conductive box; they must be in close proximity within the simulator’s box or coupled through an insulating side of the box. This coupling is shown on Fig. 1 indicated by M. To model both high and low mutual inductance scenarios, two different voltage tap connections were made: one connection contained the coupling to the main current branch, while the other did not.

The mutual inductance becomes very noticeable when using the pulse current measurement method. The magnitude of this mutual inductance was chosen to approximate typical values observed in superconductor measurements. The low-inductance lead was included in the simulator design to provide a more “ideal” test device to be used to separate possible inductive effects in the measurement system or method.

Since one function of the passive simulator is to compare the *I*_c_ results obtained from two different measurement methods, it is important to distinguish between variations in *I*_c_ due to either the intrinsic properties of the simulator or the measurement apparatus. In some situations, variations in *n* and *I*_c_ are due to the measurement apparatus. This was investigated by measuring the voltages across R2 and R3, and determining critical currents for each measurement. Since the two resistors differ by a factor of about 100, the signal magnitude was about 100 times greater at R2. Uncertainties in the correct *I*_c_ at low criteria using R3 were virtually eliminated by using the signal on R2. Thus, a lack of precision in *I*_c_ at low criteria could be attributed to the inability of the apparatus to measure low voltages accurately, not an intrinsic instability of the simulator at these signal levels.

In order to make direct comparisons between critical currents at various electric field criteria, artificial “lengths” were attributed to resistors R2 and R3. The signal strength at R2 is about 100 times greater than R3, so appropriate lengths had to be chosen to normalize the electric fields at these resistors. Since typical voltage tap separations are on the order of 1 to 100 cm on superconducting samples, the voltage taps on R2 have a length of about 100 cm associated with them, while the taps on R3 have a length of 1 cm. Thus, the “electric fields” at these voltage taps are equivalent at a given current.

An experimenter may want to use the passive simulator as a test device instead of a reference device. In this case, the simulator could be designed to have several different critical currents depending on a switch setting. Since this may lead to additional variability in *I*_c_, a reference device will most likely have only one value of *I*_c_, as well as a fuse to protect it from over-current damage.

### 2.3 Active Simulator Operation

The active simulator is not driven directly by the output of the current supply. Instead, its input is taken from the current supply’s shunt resistor. The shunt resistor serves the same function as R1 in the passive simulator. Thus, the critical current of the simulator is simply the current at which the shunt resistor reaches some target voltage. For example, if a current supply has a 50 mV shunt, then one critical current would be that current required to generate 50 mV across the shunt. Therefore, if the current required to generate 50 mV is 100 A, the critical current is 100 A. This design feature makes the active simulator a versatile component of a measurement system.

### 2.4 Active Simulator Design Considerations

**Figure 5:**
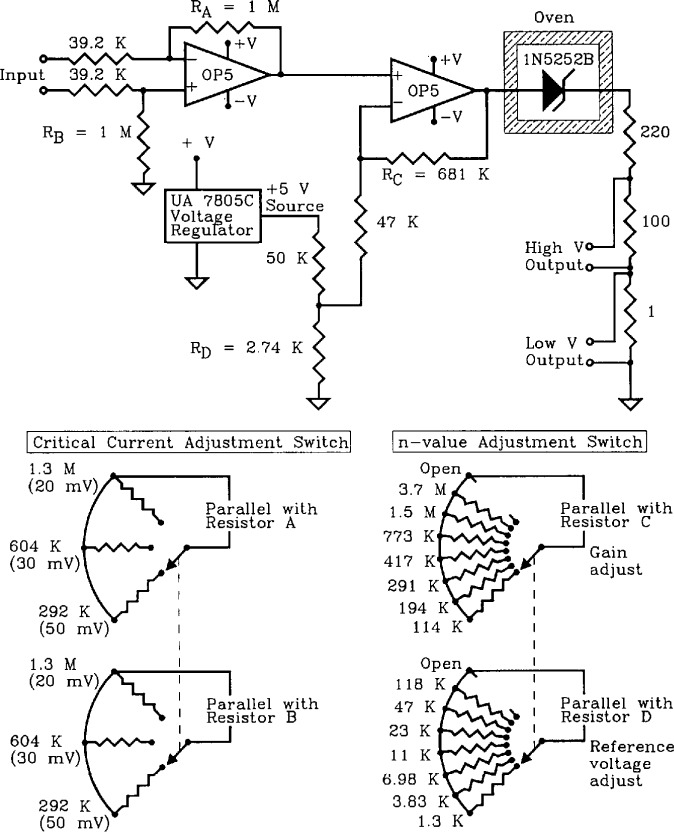
Circuit diagram of the active simulator along with diagrams of the critical-current adjustment switch and the *n*-value adjustment switch.

The active simulator does not emulate the subtle effects of a superconductor; it is meant for use as a circuit that generates various values of *n* and *I_c_.* The circuit diagram for the active simulator is shown in Fig. 5. The active simulator circuit consists of 3 stages: two cascaded operational amplifiers (op-amps), and an output stage consisting of a Zener diode and resistors that simulate the sample voltage.

The first stage of the circuit is a differential amplifier that amplifies the input signal coming from the current supply’s current-shunt resistor. To obtain different values of *I*_c_ with a given shunt resistor, the amplification of this stage is changed with a two-pole switch. Therefore, it is possible to obtain a lower (or higher) critical current for a given shunt resistor. Typical input signal values are: 20, 30, and 50 mV. The higher the amplification, the smaller the critical current.

The second stage of the circuit is a non-inverting amplifier that, in conjunction with the diode, generates different values of *n* for a given *I*_c_. The two-pole *n*-switch changes two resistance values to allow the *n*-value to be changed without significantly affecting *I*_c_. As the switch decreases the feedback resistance, the gain is decreased and thus the *n*-value is decreased.

**Figure 6:**
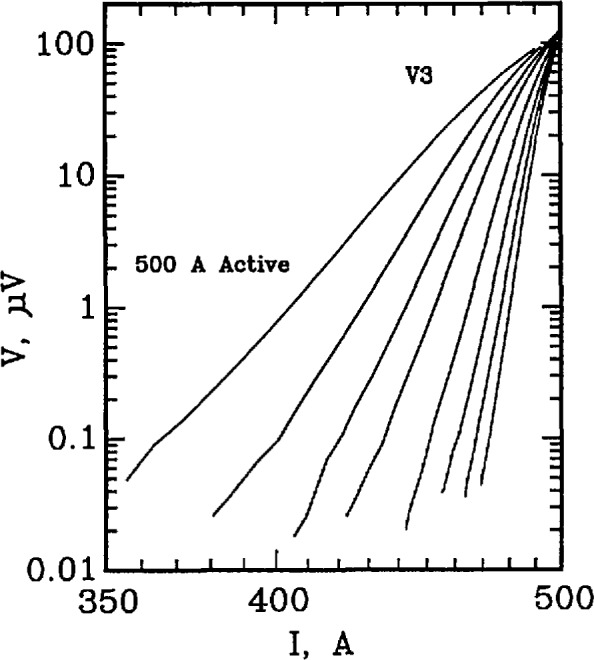
Full logarithmic plot of voltage versus current for the 500 A active simulator. Each curve corresponds to a different setting of the *n*-value switch in Fig. 3. The *n*-values range from about 25 to 144. These curves converge near 120 μV.

In order to maintain nearly the same critical current with a different value of *n*, the reference voltage of the non-inverting amplifier must be changed (the second pole of this switch). Through an iterative process, values of *n* and appropriately adjusted reference voltages were determined so that the critical currents at a given sample voltage were approximately the same. A target sample voltage of approximately 120 μV across R3 was selected. Figure 6 is a full logarithmic plot of *V* versus *I* for the active simulator for one *I*_c_ setting and 8 different *n* values. Notice that the curves intersect near the target voltage of 120 V. The eight curves in Fig. 6 correspond to the eight *n*-value adjustment switch settings on Fig. 5. The highest *n*-value (about 140) corresponds to the open switch setting.

The third stage consists of the Zener diode, and the current-limiting resistor R2, and the sample voltage resistor R3. These provide the same function as the passive simulator. Unlike the passive simulator, however, there is no provision for high mutual inductance on these voltage taps.

Table 1 indicates typical shunt resistances for the 0.5, 50, 500, and 3000 A active simulators. These resistances were calculated on the basis of a 50 mV input signal. The input terminals of the active simulator are connected across the shunt resistor. The values of R4 are not applicable here, since discrete resistors were used instead of distributed wire resistors.

## 3. Data Acquisition and Analysis

The superconductor simulators described here can be used to establish the integrity of a superconductor data acquisition system. This section gives a brief overview of the acquisition system that was used in conjunction with these simulators. This information is given to specify the way that these simulators were characterized. It is by no means the only system that can be used with the simulators. For a full discussion of various superconductor measurement systems, consult Refs. [3,5].

Measurement systems can vary in complexity from a simple analog recorder that monitors the voltage-current characteristic, to a sophisticated computerized data-acquisition system that monitors several additional experimental parameters. The choice of measurement system depends on considerations such as the number of samples to be measured, accuracy and precision requirements, and the number of experimental parameters that need to be monitored.

### 3.1 Data Acquisition System

One motivation for designing the simulators was to determine the integrity of the data-acquisition system described here, and to study the effects of current ripple on superconductor measurements [6]. This system is computer controlled and relies on analog or digital voltmeters^1^ to make voltage and current measurements. The system is capable of monitoring magnetic field, sample temperature, and other important experimental parameters.

The step-and-hold-current method was used to determine the critical current of the superconductor simulator. As the name suggests, the sample current is abruptly increased to a preset level, and held at that level while the sample voltage and current are measured. From that level, the sample current is again abruptly increased to another preset level and held. This cycle is repeated until a preset current limit or voltage limit is reached.

The preset current levels, or current setpoints, are determined to maximize the characterization of the entire *V-I* curve. The curve is divided into two regions: a low-current/low-voltage region, and a high-current/high-voltage region. In the low-current region, the setpoints are chosen to be equally spaced on a linear scale. In the high-current regions, the current setpoints are chosen to correspond to voltages that are equally spaced on a logarithmic scale. The current setpoint that divides the low-current region from the high-current region corresponds to a sample voltage that is just below the voltage noise level.

Table 2 contains current setpoints for *n*-values of 25, 58, and 122. These setpoints are listed as percentages of *I*_24_, which is the current at the 24th setpoint. This current corresponds to a guessed value of the maximum current.

Setpoints 1 through 6 in Table 2 correspond to the low-current region of the *V-I* characteristic, and are equally spaced on a linear scale. Setpoints 7 and 8 correspond to the transition region from low currents to high currents. Setpoints 9 through 24 correspond to equally spaced voltages on a logarithmic scale. Setpoint 25 is for a zero current measurement of voltage for thermal corrections. This pattern is illustrated on Figs. 2, 3, and 4. The symbols along the curves indicate the individual setpoints. The lowest non-zero current point deviates from this pattern because of the turn-on characteristics of the current supply. Otherwise, the points illustrate this pattern. The 24th point is off the scale of Figs. 2 and 3.

To determine the critical current after a *V-I* curve is obtained, an electric field criterion is applied to the characteristic. For a complete discussion of critical current measurement methodology, consult Refs. [1,3].

### 3.2 Role of the Simulator in Data Acquisition Systems

In general, the more sophisticated the acquisition system, the more testing it must undergo to establish its integrity. Thus, the computer-controlled system discussed above must be exhaustively tested.

The simulator is an idealization of a superconductor’s electrical characteristics: the *V-I* curve, thermoelectric voltages, and mutual inductances. It can therefore be used to test a measurement system’s integrity under various experimental conditions. For example, to determine the integrity of the measurement system under conditions of high mutual inductance, an experimenter would use the high inductance terminals of the simulator; for high thermoelectric noise conditions, a lead-shot bag could be removed from the voltage-tap terminals. Similar tests can be performed for low-inductance conditions and other experimental phenomena.

The idealization of the superconductor simulator enables the experimenter to understand the limitations of a particular measurement system and method. With a standard reference material, these limitations may be misinterpreted as properties of the superconductor instead of the measurement system.

Although the simulator provides an expedient test for the measurement system, it does not test the magnet, cryostat, temperature controller, or other similar devices. A standard reference material is best suited for testing these components. There are events such as sample motion, contact heating, early quenches, and irregular or unstable *V-I* characteristics that are not emulated by these simulators. Such phenomena may be emulated in future designs.

### 3.3 Systematic Errors in Data Analysis

The empirical model of a superconductor’s *V-I* characteristic given in Eq. (1) is a simple approximation to the actual characteristic. Systematic errors arise from this approximation, and could be avoided by using a more sophisticated model. For example, a least-squares power-series representation of the *V-I* characteristic could be used as long as there are not too many adjustable parameters.

Perhaps the least realistic aspect of the empirical model is the constancy of *n* in Eq. (1). In general, *n* changes along the *V-I* curve of a real superconductor, so a more sophisticated model would take this into account. The approach used in this analysis routine to allow for variations in *n* is to divide the non-zero portion of the *V-I* curve into overlapping intervals, fitting the *V-I* points in each interval, and calculating a value of *n* for each interval. Thus, to determine *I*_c_ at various criteria, one would use the fit parameters that corresponds to that criterion.

The intervals that were used with these simulators are given in Table 3. The first column of Table 3 contains the electric field criterion that would be used to determine a critical current. The following two columns contain the upper and lower limits of the electric field window. The initial and terminal points of each interval are somewhat arbitrary in this model. These values are not necessarily optimal, but are included here for completeness. They were selected based on the noise level of the system and on a compromise between accuracy and precision. The last column indicates the ratio of the upper limit to the lower limit. This ratio monotonically decreases with increasing electric field criterion, in order to include more data points in electric field intervals where the signal-to-noise ratio is small.

The critical current at a specified electric field is determined by interpolating the current to that criterion using the appropriate value of *n* and the constant *V*_o_/(*I*_o_)*^n^*. Systematic errors were found to be on the order of 0.1% at a criterion of 0.1 μV/cm when using this method. At criteria of 1 μV/cm and above, these errors were almost undetectable. In order to increase precision in *I*_c_ determinations, more points along the *V-I* curve could be used, but that would increase the systematic error since then a larger portion of the curve would be used in the calculation. This tradeoff must be taken into consideration when developing data analysis software.

Another technique to more accurately characterize the *V-I* curve is to perform a polynomial fit in *n.* An expression of this form would characterize the *V-I* curve, but it may not be applicable for a range of materials, temperatures, or magnetic fields.

## 4. Superconductor Simulator Performance

A systematic study of various electrical characteristics of the passive, active, and hybrid simulators was performed, and the results are shown below. These studies included a characterization of *I*_c_ versus temperature, *I*_c_ versus time, *n* versus *V*, effects of inductance on *I*_c_, and effects of current magnitude and current ripple on simulator characteristics.

### 4.1 Temperature Dependence of *I*_c_

**Figure 7:**
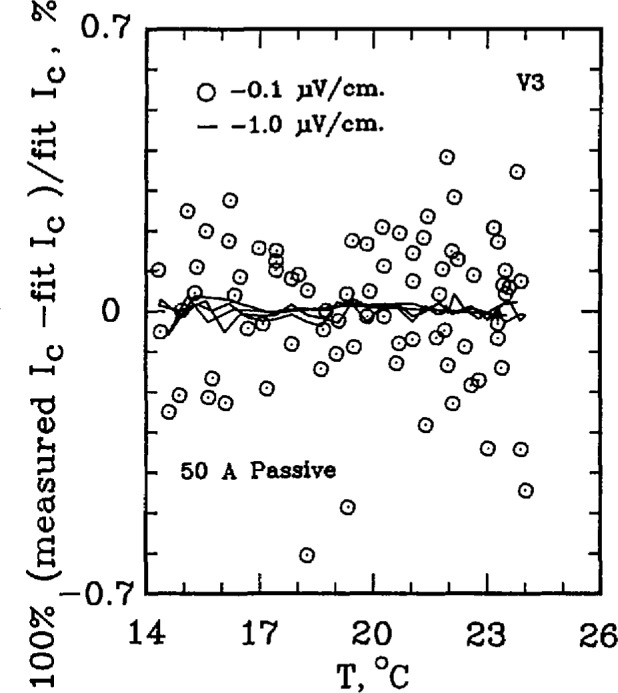
Difference from linear temperature fit for the SO A (*n* =25) passive simulator. Data obtained using R3, 80 points used in the fit.

**Figure 8:**
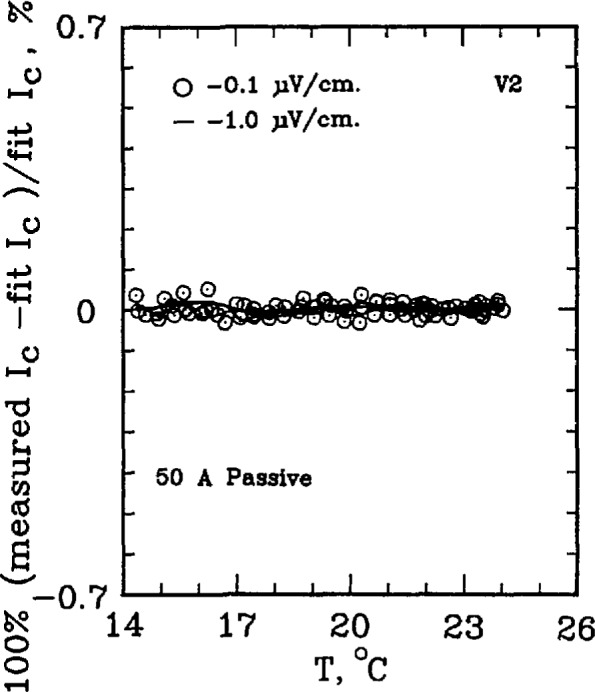
Difference from linear temperature fit for the 50 A (*n*≈25) passive simulator. Data obtained using R2, 80 points used in the fit.

The critical current of a superconductor is a strong function of temperature. For example, for the standard reference material SRM 1457 (Cu/Nb-Ti), a 21.2% change in *I*_c_ per K is observed at an applied field of 2 T. At higher fields, this temperature dependence is amplified. For example, at 8 T, the temperature coefficient is approximately 62% per K. This level of temperature dependence makes testing the measurement system with the SRM a challenging task. The superconductor simulator, however, has a temperature coefficient on the order of 0.3% per K. Figure 7 shows the percent difference of the measured critical current on R3 from a linear fit of critical currents over a temperature interval of 10 °C near room temperature. As this figure indicates, most of the 0.1 μV/cm data lies within ±0.4% of the fit line and within ±0.1% for 1.0 μV/cm. Higher electric field criteria have even less variability. If the critical currents were measured on R2, the uncertainty reduces to less than ±0.1% of the fit line for all electric field criteria, indicated on Fig. 8. Thus, the simulator is superior to the SRM for testing the system for repeatability in *I*_c_ measurements, even after taking the variability in room temperatures and liquid helium temperatures into account.

Tables 4 and 5 show critical-current temperature coefficients for the active, passive, and hybrid simulators at various electric field criteria as characterized by:

$$I_{c}=b+m\cdot T$$ (3)

where *I*_c_ and *b* are in A, *m* is in A/°C, and *T* is in °C. Table 4 contains data for the active simulator, while Table 5 contains data for the passive and hybrid simulator. Each linear fit used between 40 and 120 points. Each data set contained points taken with both increasing and decreasing temperature. There was no significant hysteresis with direction of temperature sweep for temperature rates-of-change as large as 3 °C/h. The temperature range of each set was not identical, but they were all near room temperature. Because the temperature range did not include 0 °C, comparisons of *I*_c_ were not made 0 °C (using just *b*), but at a temperature of 23 to 26 °C. The first and second columns identify the simulator and typical electric field criteria, while the following two columns give values of *b* and *m* at those criteria. The 5th and 6th columns show the standard deviation from the fitted line for R2 and R3, respectively.

Table 4 also contains data showing the active simulator’s performance with different critical current ranges. For an *n*-value of 58, critical currents of 0.2, 0.3, 0.5, 50, 500, and 3000 A were obtained, along with the temperature fit parameters *b* and *m* for each electric field criterion. The active simulator has an almost negligible temperature dependence, on the order of 0.007% Δ*I*_c_/°C at 1 μV/cm. For a given power supply, different critical currents can be obtained by changing the gain of the first stage of the active simulator circuit.

Although the current characteristic of a diode is exponentially related to temperature, a linear approximation is appropriate since small temperature swings are being considered about a quiescent temperature of 23 °C. No significant departure from linearity was observed for temperature ranges of approximately 10 °C.

For a given diode, the temperature fit parameters *b* and *m* are linearly related to the rated critical current. In Table 5, for example, the 50 A passive simulator and the 2 A passive simulator have a ratio of m-values (and 6-values) of approximately 25, which is the ratio of the rated critical currents. Since the critical currents are inversely related to the input resistor R1, this ratio scales inversely with R1.

Table 5 also shows that the temperature dependence of the passive simulator is small. For example, for the 2 A passive simulator at a signal level of 1 μV/cm, the temperature dependence is on the order of 0.27% Δ*I*_c_/°C. For the hybrid simulator, this dependence is less than 0.001% Δ*I*_c_/°C. The hybrid simulator has a lower temperature dependence than that of the active simulator because the active simulator has more components (operational amplifiers, resistors) outside the temperature controlled oven.

The last column shows a percentage difference in critical current between the voltages at R3 and R2, normalized with respect to the voltage at R2. At higher electric field criteria, this difference is significantly reduced. For the passive and hybrid simulators, the difference in *I*_c_ at 1 μV/cm between V3 and V2 was about 0.01%. These values were generally less than the standard deviation of the temperature dependence of V3 about the fit line. For the active simulator, these differences were typically 0.005%. This smaller value is due, in part, to the higher value of *n* for the active simulator.

**Figure 9:**
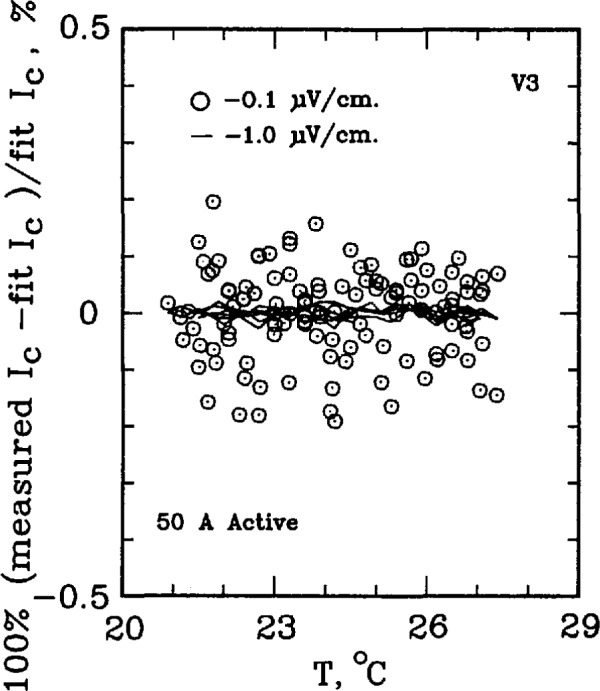
Difference from linear temperature fit for the SO A (*n*≈58) active simulator. Data obtained using R3, 120 points used in the fit.

**Figure 10:**
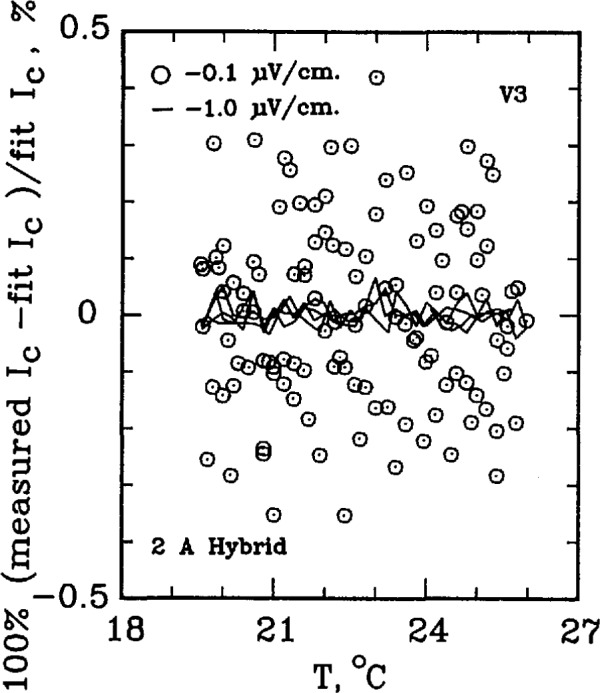
Difference from linear temperature fit for the 2 A (*n*≈25) hybrid simulator. Data obtained using R3, 120 points used in the fit.

Figures 9 and 10 show the percent difference of the measured critical current on R3 from a linear fit of critical currents over a temperature interval of 6–7 °C for the 50 A active simulator and the 2 A hybrid simulator. The data scatter on Fig. 9 is slightly less than that in Fig. 10 because of the higher *n*-value of the active simulator: the active simulator is set at *n* =58 for Fig. 9, whereas the hybrid simulator has an *n* of 25 in Fig. 10. This lower scatter is due to the fact that the same voltage error results in a smaller change in *I*_c_ for a curve with a higher *n*-value.

This is further illustrated in the active simulator data shown in Table 4. The first three sets in Table 4 have the same *I*_c_ but three different *n*-values: 25, 58, and 122, respectively. The coefficients of variation reduce with increasing *n.* Thus, the higher the *n*-value, the greater the precision possible in critical-current measurements.

### 4.2 Low-Voltage Data

An experiment was performed to establish the precision and accuracy of the simulator at lower electric field criteria. A comparison of the performance of analog and digital voltmeters was also made.

The dual-voltage-tap feature implemented in these simulators has a broad range of applications. For example, these taps could be used to compare the measurement precision of analog and digital voltmeters. In this experiment, the analog meter could be connected to the low voltage taps R3 (voltage signal V3), and the digital meter to the high voltage taps. Because of the relative signal size, a less sensitive device can be used on R2 (voltage signal V2). This experiment was conducted, and the resultant data is shown in Table 6. The analog voltmeter was able to accurately measure small voltages with an uncertainty of 2 nV (standard deviation about 3 nV). The digital voltmeter uncertainty is on the order of 10 nV (standard deviation about 27 nV).

Tables 6 and 7 show the temperature coefficients for the 50 A passive simulator at low electric field criteria. Table 6 contains data which were taken using a digital nanovoltmeter on R2 (the high voltage tap, V2), and an analog nanovoltmeter on R3 (low voltage tap, V3). The data on Table 7 were taken using digital nanovoltmeters on both taps. Comparing the data taken on R3 for the digital voltmeter and the analog voltmeter shows that the coefficients of variation differ by a factor of 3. This is not as large as the ratio of the noise levels of the two meters because the method of curve fitting reduces the effects of noise. The accuracies indicated by the Δ*I*_c_ in the last column are about the same for the two systems. This indicates that higher noise levels do not severely compromise accuracy. With signals of 1 μV or more, the digital meter and the analog meter have similar responses.

### 4.3 Stability of *I*_c_ over Time

The stability of the simulators’ characteristics is crucial for their use as reference devices. Thus, experiments were performed in order to quantify any differences in critical current over time. Table 8 shows the results of these experiments. The blank spaces in this table indicate data that were unavailable. The change in critical current over a period of 56 d was less than 0.02% for the 2 A hybrid simulator at an electric field criterion of 1 μV/cm. The change in critical current over a period of 106 d was about 0.01% for the 2 A passive simulator at an electric field criterion of 1 μV/cm. Only the critical current of the active simulator seemed to have a finite drift with time at a rate of about 0.016%/month.

The stability of thermoelectric offset voltages with time was characterized, since this can affect the *I*_c_. The passive simulator typically developed an offset voltage of less than 10 nV during the time (~2 min) of a dc measurement of *I*_c_. This is typical of the thermoelectric voltages observed during superconductor testing.

### 4.4 Effect of Power Supply on Critical Current

The active simulator was designed for use with any dc power supply. Table 9 compares critical currents and their associated shunt voltages for the 50 mV active simulator. All data were taken on R2, with an electric field criterion of 10 μV/cm. The equivalent shunt voltage in this table refers to the voltage across the power supply’s input resistor at the rated critical current. Thus, regardless of the actual critical current, the shunt voltage (and hence the input signal to the active simulator) stays within 0.06% of 47.124 mV except for the measurements using the high-current power supply. Most of this 0.06% is a result of the small drift in *I*_c_ with time mentioned above.

The larger capacity power supplies exhibited a greater percent difference from the 10 A supply. This is due to the effects of current ripple in these supplies, and is reduced with the introduction of current-ripple filtering, as is shown on the last row of Table 9.

The introduction of current ripple changes the determined critical current. Due to the voltage-current relationship described by Eq. (1), the greater the *n*-value for a given modulation, the greater the reduction in the determined critical current. The reduction in critical current at a given electric field criterion is a function of the *peak* value of the current ripple, as opposed to the rms value. These effects have been extensively studied, and are discussed in Ref. [6].

Since current ripple is more prevalent in higher-current power supplies, an active current filter was designed and installed in a 3000 A power supply. Current filtering is performed by inverting the ripple component of the signal and adding the inverted component back to the power supply’s original signal. In this manner, the current ripple can be reduced by a factor of 8. Typically, the 3000 A power supply’s ripple has a peak of 37 A without active filtering. With the active filter, the peak value of ripple is reduced to approximately 4.5 A. A detailed discussion of the 3000 A active current filter of the power supply is given in Ref. [7]. The effect of current ripple in this simulator is quantitatively similar to the effect on a superconductor. According to the models in [6], a peak ripple of 37 A on a direct current of 2819 A in a superconductor with an *n*-value of 58 would reduce *I*_c_ by about 0.3%. These results were in close agreement. The effect of 4.5 A peak ripple should have been about 0.04%, but was slightly larger, at 0.10%. This takes into account the drift of *I*_c_ with time. This extra effect may be due to the small spikes from the SCR (silicon-controlled rectifier) that are still present on the supply. The 10 A and the 1 kA current supplies have extremely low current ripple and no SCR spikes. Circuit details for these two supplies are given in Refs. [8,9], respectively.

### 4.5 Comparison of *n*-Value between the Simulator and Superconductors

The characteristics of the simulator are comparable to those of both low-*T*_c_ and high-*T*_c_ superconductors. Typically, *“n”* in Eq. (1) is used as a quality index for the superconductor. The higher the *n*, the greater the superconductor filament homogeneity. The active simulator can generate *V-I* characteristics with a wide variety of *n’s.* For a complete discussion of the quality index *n* for superconductors and the shape of the *V-I* curve, consult Refs. [10,11,12].

**Figure 11:**
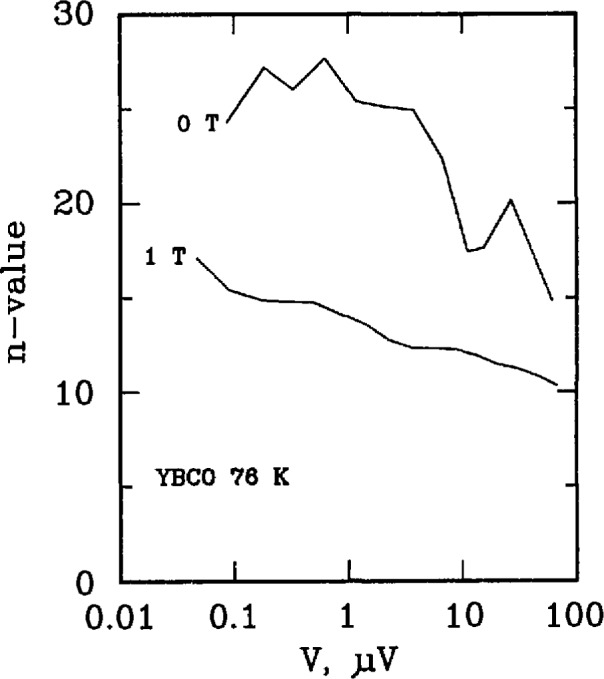
Plot of *n*-value versus log voltage for a YBCO superconductor at 76 K at magnetic fields of 0 and 1 T. The critical currents ranged from 2 to 0.2 A, the voltage tap separation was 0.5 cm, and the area of the superconductor was 1.5 × 10^−6^ cm^2^.

**Figure 12:**
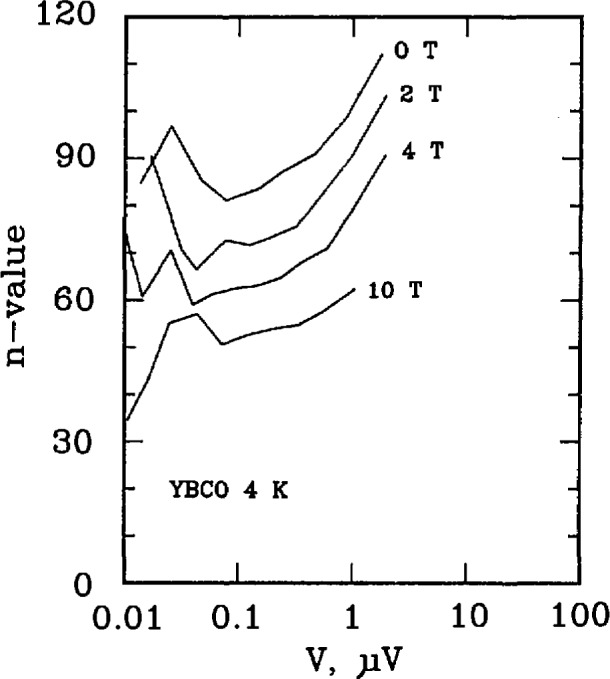
Plot of *n*-value versus voltage for a YBCO superconductor at 4 K at magnetic fields of 0, 2, 4, and 10 T. The increase in *n*-value above 1 μV is due to sample heating. The critical currents ranged from 5 to 4 A, the voltage tap separation was 0.3 cm, and the area of the superconductor was 3 × 10^−7^ cm^2^

**Figure 13:**
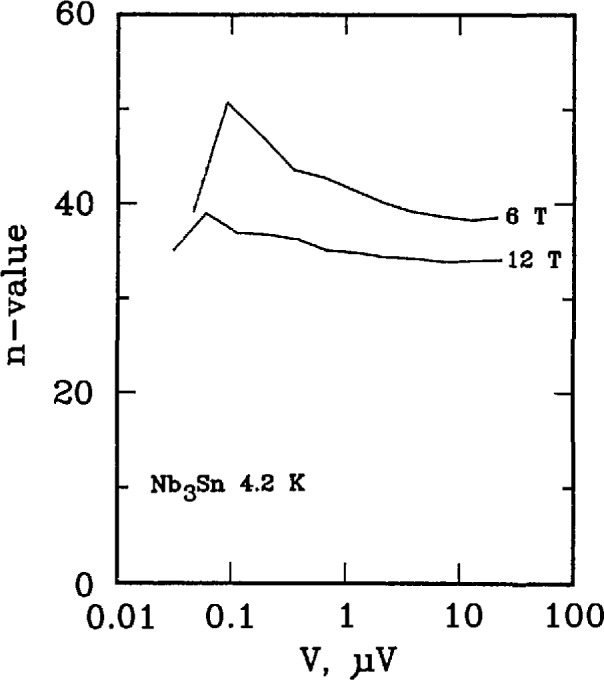
Plot of *n*-value versus voltage for a Nb_3_Sn superconductor at magnetic fields of 6 and 12 T. The critical currents ranged from 500 to 185 A, the voltage tap separation was 16 cm, and the area of the non-copper was 2.9 × 10^−3^ cm^2^.

**Figure 14:**
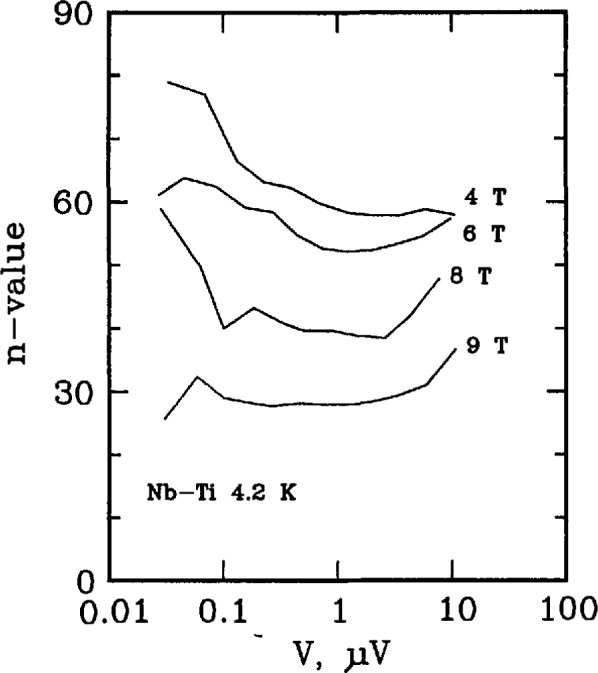
Plot of *n*-value versus voltage for a Nb-Ti superconductor at magnetic fields of 4, 6, 8, and 9 T. These data were taken on the standard reference material SRM 1457. The critical currents ranged from 200 to 45 A, the voltage tap separation was 15 cm, and the area of the superconductor was 8.3 × 10^−4^ cm^2^.

**Figure 15:**
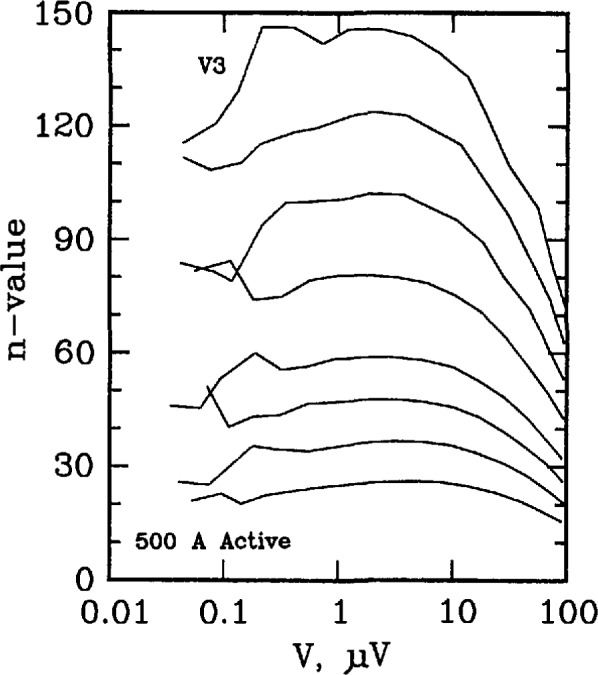
Plot of *n*-value versus voltage signal V3 for the 500 A active simulator.

Three types of superconductors were tested: YBCO thin film, Nb_3_Sn, and Nb-Ti. The YBCO is a high-*T*_c_ sample, and the latter two are law-*T*_c_ samples. Figures 11 and 12 show the *n* versus log voltage plots for a YBCO sample at 76 and 4 K, respectively. Figures 13 and 14 are *n* versus voltage plots for Nb_3_Sn and Nb-Ti superconductors. The Nb-Ti sample is the standard reference material SRM 1457. Figure 15 plots *n* versus voltage for the 500 A active simulator. These eight curves correspond to the eight *n*-value adjustment switch settings on Fig. 5. At voltages below 0.1 μV, the variation in *n* is due to limitations in voltage measurements.

Figure 11 plots *n*-versus voltage for YBCO at 76 K and fields of 0 and 1 T. All measurements were made using the dc method. These values of *n* lie between approximately 10 and 30. Figure 12 plots *n* versus voltage for a YBCO sample at liquid helium temperatures and at four magnetic fields, 0, 2, 4, and 10 T. These values of *n* can be as high as 100.

Figures 13 and 14 show *n* values between 30–50 for Nb_3_Sn (6 and 12 T), and 40–90 for Nb-Ti (4, 6, 8, 9 T), respectively. The increase in value of *n* at high voltages on Figs. 12 and 14 is due to slight sample heating.

Figure 15 plots *n* versus voltage for the active simulator using measurements on R3. In this plot, *n* was varied using the switch between values of 25 and 150. In the region between 0.1 and 10 μV, the values of *n* remained relatively constant, as in the case of superconductors. Thus, with this simulator, an experimenter can emulate a substantial range of values of *n* that are observed in superconductors. In Fig. 15, the highest *n*-value corresponds to an open circuit setting on the *n*-value switch on Fig. 5.

**Figure 16:**
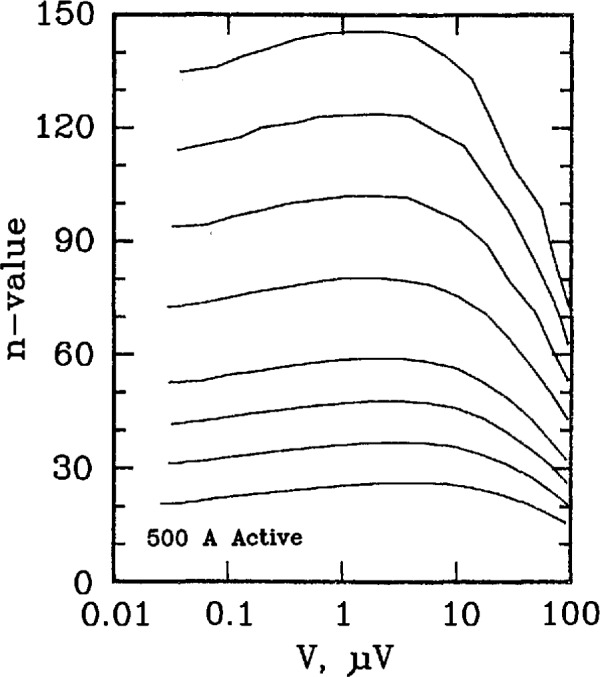
Plot of *n*-value versus voltage signal V3 for the 500 A active simulator using measurements on R2 (higher voltage signal, V2).

Figure 16 plots *n* versus voltage for the active simulator using voltage measurements on R2 (higher voltage signal). Values of *n* were inferred from these measurements and were plotted against the corresponding voltage, V3. This was done to illustrate that the actual *n* of the simulators at low voltages is still a well-behaved function of voltage. The plots of *n* versus voltage after this were also inferred from R2.

**Figure 17:**
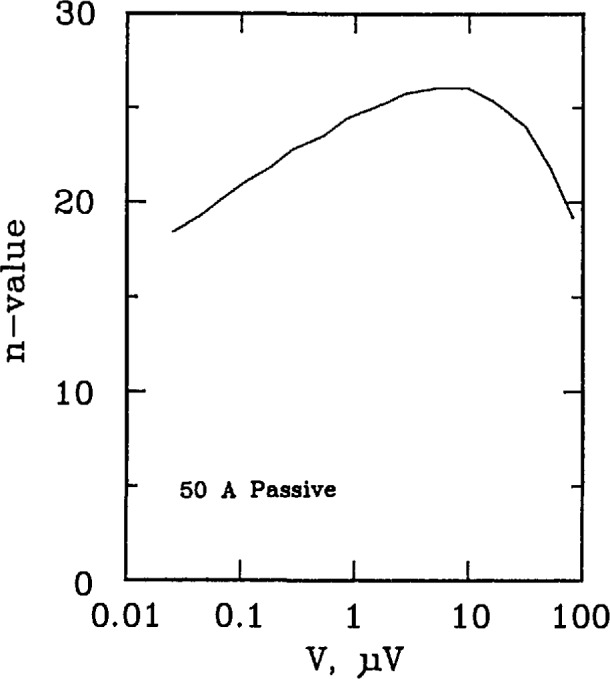
Plot of *n*-value versus voltage for the 50 A passive simulator.

**Figure 18:**
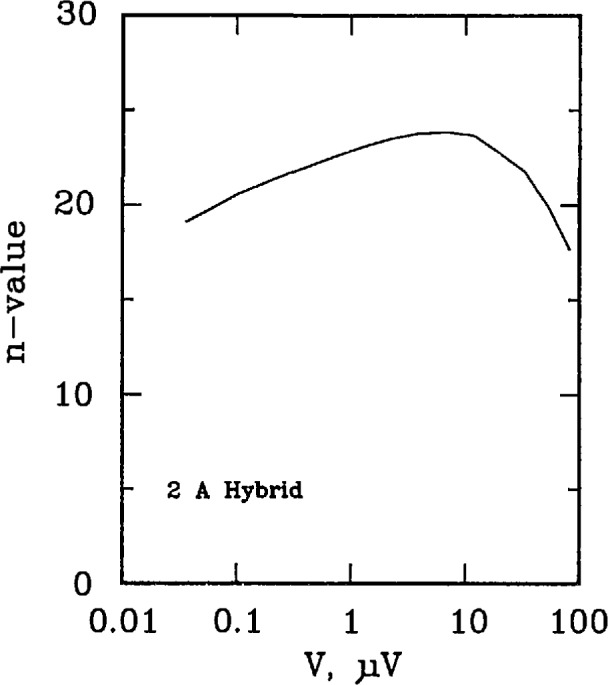
Plot of *n*-value versus voltage for the 2 A hybrid simulator.

Figures 17 and 18 plots the *n* versus voltage for the 50 A passive simulator and the 2 A hybrid simulators. Each curve has a peak *n*-value of approximately 24. The shapes of these curves are representative of those taken on a superconductor if sample heating is not a problem.

### 4.6 Discussion of Inductance

The mutual inductance between the superconductor and the voltage taps was emulated in the passive and hybrid simulators. In both a superconductor and the simulator, the mutual inductance is approximately 2 nH for voltage taps separated by a few cm. Although this inductance may seem negligible, at ramp rates of 200,000 A/s it translates to a voltage of approximately 400 μV. Ramp rates of this magnitude are typical when using the pulse current technique to measure critical current.

**Figure 19:**
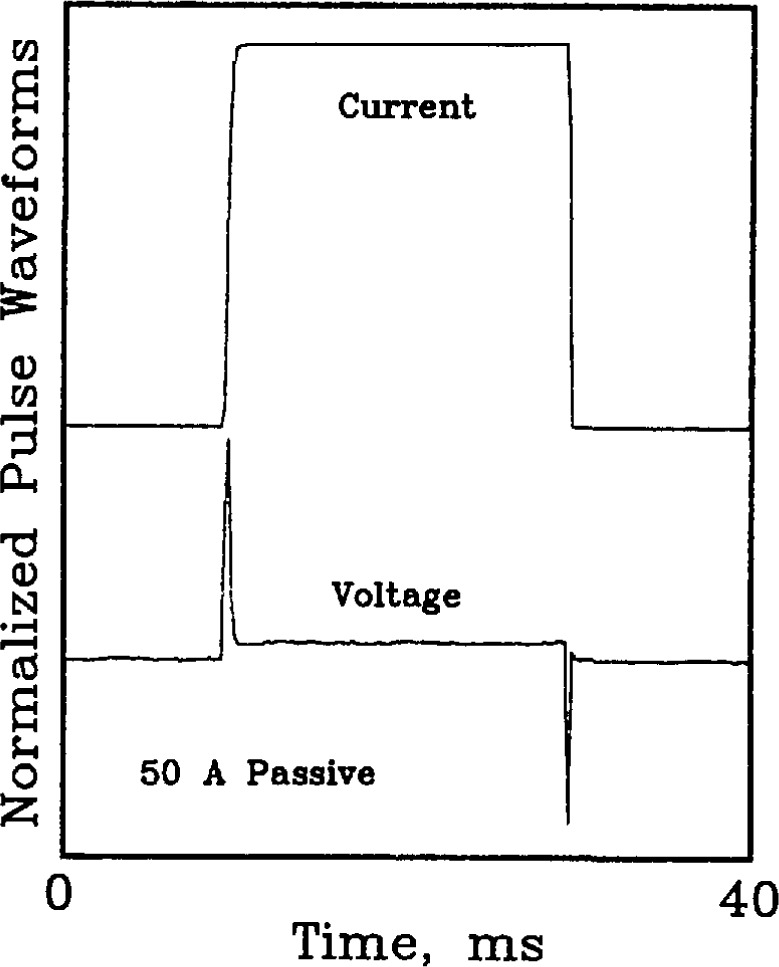
Current and resultant voltage pulses delivered to the 50 A passive simulator. Maximum current=48.1 A, peak voltage = 180 μV, steady state voltage drop = 20 μV, frequency band pass=dc to 10 kHz. Positive and negative voltage spikes correspond to positive and negative d*I/*d*t.*

**Figure 20:**
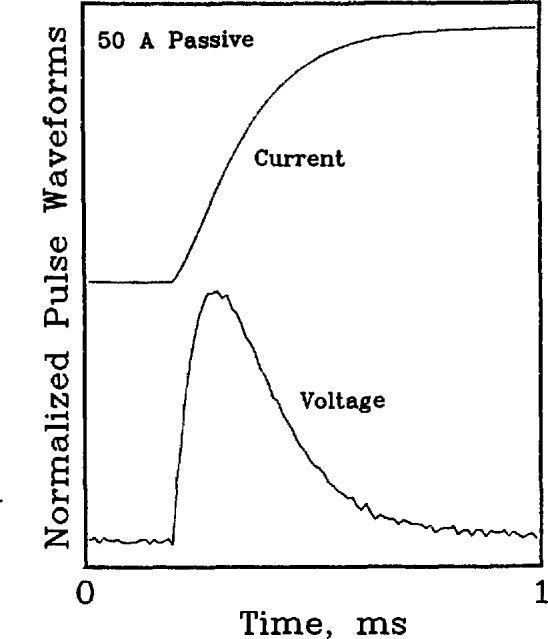
Enlargement of the leading edge of a current and resultant voltage pulse. Maximum current=45.6 A, maximum d*I*/d*t* = 188,000 A/s, peak voltage = 400 μV, frequency band pass=dc to 100 kHz.

Figures 19 and 20 are plots of the voltage wave-form resulting from delivering a current pulse to the passive simulator. Figure 20 is an enlargement of the leading edge of the current and voltage pulses. The voltage spikes at the leading and trailing edges of the voltage waveform on Fig. 19 are due to the mutual inductance of the simulator. The voltage spikes peak near the peak d*I*/d*t.* The polarity of the induced voltage with positive d*I*/d*t* is the same as the polarity of the resistive transition of the superconductor. This polarity was chosen to test the acquisition and analysis system. The amount by which the measured voltage was different from zero in the low-current portion of the *V-I* characteristic (where the actual voltage is zero), would indicate the existence of a problem with the induced, ground-loop, or common-mode voltages.

## 5. Discussion

The superconductor simulators discussed here represent an important step in diagnostic test equipment for superconductor measurement systems. These devices can aid the experimenter in determining sources of systematic errors in data analysis routines, data acquisition systems, and other problems in the measurement system. Currently, the simulators emulate the major electrical characteristics of superconductors. In the future, however, the simulators could have additional features added to make them more comprehensive test devices.

Some additional features that could be added to the simulators include a button to increase electrical noise at the voltage taps, current-transfer emulation, simulation of a sample quench, and shifting *V-I* characteristics.

Electrical noise could be incorporated in the active simulator by introducing a noise generator at the voltage taps. When the appropriate button is pressed, the noise generator signal could be combined with the output voltage. For a passive simulator, instead of a noise generator, an antenna could be used to increase the signal noise level.

The addition of the noise generator signal to the output signal could be done at two locations in the circuit: at the input junction (before the nonlinear circuit elements), or at the output stage (after the nonlinear elements). Inserting the noise component before the nonlinear elements would result in a nonlinear amplification of the noise at the output stage, whereas implementing the noise at the output stage would result in an increase in the quiescent noise level. Current ripple from the current supply in a superconductor can be best emulated by adding the noise signal at the input stage. Either of these implementations may be appropriate depending upon what test is being performed.

To simulate current transfer in the simulator, a large resistor (approximately 25 kΩ) should be added in parallel with the diode. With this addition, the current transfer corrections in the data analysis routines could also be tested.

Sample quenches could be implemented in the simulator by installing a quench detector (a device that interrupts current flow in the event that the sample voltage exceeds a preset voltage-trip point) across resistor R1, and setting the voltage-trip point near or below 0.7 V. Thus, as the current is increased through the simulator, it will be interrupted before the diode starts conducting current, and a null voltage-current characteristic would be obtained.

A “shifting” voltage-current characteristic could be obtained by temporarily short-circuiting resistors R2 and/or R3 or the diode. In this manner, the sample voltage would seem to shift with a steady current ramp.

The overall quality of the active simulator could be improved by exchanging the operational amplifiers in the circuit with instrument-quality amplifiers. Although this would increase the cost of the simulator, the stability, linearity, frequency response, and input impedance of the simulator would probably be favorably affected.

### 5.1 Uses of the Simulators

The applications for the superconductor simulator range from use as a training aid to a device used in the development of new test methods, to a standard test device used to characterize and identify sources of error within a measurement system. For example, simulators are useful for training experimenters who lack experience in making superconductor measurements. Instead of using expensive superconductor samples, and expending cryogens, the simulators can be used many times at room temperatures. Thus, inexperienced experimenters can practice measuring the critical current of the simulator and can familiarize themselves with the test apparatus.

Experimenters already familiar with superconductor measurements can use the simulators to develop new measurement systems and methods without consuming actual samples and their associated high costs. The stability of the simulator’s *V-I* characteristic over time allows the experimenter to determine sources of variation between the new measurement scheme and standard schemes. The superconductor simulator could be used as a standard test device for interlaboratory comparison of measurement systems and methods. The active and the passive simulators can be used as test devices for the measurement apparatus and measurement method. The simulators can aid in identifying common-mode voltages and ground loops in the pulse method of critical current determination since the lower currents should have no voltage drop.

The accuracy of the simulator is limited by the accuracy of the current-sensing resistors, voltmeters used for current and voltage measurements, and the accuracy of the measurement of room temperature or diode temperature. One distinct advantage of the hybrid and active simulators is that the accuracy of the temperature measurement is not as significant as it is for the passive device. It should also be noted that the calibration of the current-sensing resistor is not tested by the active simulator because of the way in which it gets its input signal. For these reasons, the hybrid simulator may be the best candidate for use as a reference device to compare measurements. The total uncertainty of the hybrid simulator is estimated to be about ± 0.2% at 1 μV/cm with the uncertainty of the current sensing resistor being the major source of uncertainty.

## 6. Summary

The superconductor simulator’s electrical characteristics have been extensively investigated. The salient points of these studies are given below:

*General Features:*
  - The simulators can test the integrity of a complex measurement system.
  - These simulators exhibit a small temperature dependence, and a small drift with time (less than 0.06% Δ*I*_c_ in a 3 month period).
  - The variability of these simulators is typically less than 0.1% at a voltage of 1 μV, Large values of *n* or greater signal levels reduce this variability.
  - Each simulator is equipped with two sample voltage taps: R2 passively generates a voltage that is approximately 100 times greater than the signal at R3. The simulators are well behaved at voltages below 0.01 μV.
  - Each simulator’s response to current ripple is comparable to that of a superconductor.
*Passive Simulator:*
  - Can be used as a superconductor sample substitution box.
  - Small number of passive, low-reactance components.
  - Critical currents: 2, 25, 50 A.
  - *n*-value: 25.
  - Temperature dependence: less than a superconductor, typically about 0.3% Δ*I*_c_/°C at a voltage level of 1 μV.
*Hybrid Simulator:*
  - Can be used as a standard reference device.
  - Circuit identical to that of the passive circuit, except the Zener diode is enclosed in a temperature controller.
  - Critical current: 2 A.
  - *n*-value: 25.
  - Temperature dependence: typically about 0.001% Δ*I*_c_/°C at a voltage level of 1 μV.
*Active Simulator:*
  - Best suited for generating *V-I* characteristics with a wide range of *n*-values and critical currents.
  - Circuitry contains active components such as operational amplifiers.
  - Critical currents: 0.5 to 3000 A.
  - *n*-value: 25 to 150.
  - Temperature dependence: typically about 0.007% Δ*I*_c_/°C at a voltage level of 1 μV.

## 7. Conclusions

The superconductor simulator represents a simple answer to the problem of testing a complex measurement system. The simulator does not test effects of sample mounting, magnetic field, temperature control, or other similar experimental factors. However, it isolates effects solely due to the measurement system from all of the effects that can lead to variability in critical current measurements. The exploitation of its capabilities can make a dramatic difference in characterizing the current carrying capacity of low-*T*_c_ and high-*T*_c_ materials. It allows the experimenter to understand the limitations of a measurement system and the factors that can create errors in the measurement. In many modern computer controlled data acquisition systems, it is imperative that the system and analysis routines be thoroughly tested. The simulators discussed here provide such a test facility.

The passive simulator is the simplest to construct and has the fewest components, and is adequate for some applications. The hybrid simulator has the same circuitry as the passive simulator, with the addition of a temperature control oven for the diode. The hybrid simulator is best suited for use as a reference device. The active simulator is a complicated circuit with a large number of components. It is not a sample substitution box, but it is the most versatile simulator.

The simulators are a superior technology for testing the integrity of superconductor measurement systems, since they offer significantly higher precision and accuracy. The temperature coefficient for the simulators is less than 0.3% Δ*I*_c_/°C for the passive simulator and about 0.007% Δ*I*_c_/°C for the active simulator and about 0.001% Δ*I*_c_/°C for the hybrid simulator at a 1 μV signal level. The standard reference material SRM 1457, on the other hand, has a temperature coefficient of 21% Δ*I*_c_/K at a magnetic field of 2 T, and does not offer the features of stability or multiple use feature that the simulators do. The temperature coefficient of the simulators is significantly less than that of the SRM, even after considering the relative variability of liquid helium and room temperatures. The total uncertainty of the hybrid simulator is estimated to be about ± 0.2%, with the uncertainty of the current sensing resistor as the major source of this total uncertainty. This is significantly less than the uncertainty of the present superconducting reference material, which is ± 1.71 to ±2.57%, depending on the magnetic field.

## Figures and Tables

**Table 1:** Resistance values for the simulators

Simulator	Input *R*, mΩ	R1, Ω	R2, Ω	R3, mΩ	R2/R3	R4, Ω
Passive, 2 A	N/A	0349	3.12	31.3	99.36	2.53
Passive, 25 A	N/A	0304	3.25	32.4	100.29	2.27
Passive, 50 A	N/A	0.160	3.25	32.4	100.29	2.27
Hybrid, 2 A	N/A	0.347	3.49	34.6	100.84	2.09
Active, 0.5 A	100.	N/A	100.2	1004	99.75	N/A
Active, 50 A	1.00	N/A	100.2	1004	99.75	N/A
Active, 500 A	0.100	N/A	100.2	1004	99.75	N/A
Active, 3000 A	0.0167	N/A	100.2	1004	99.75	N/A

**Table 2:** Target voltages and current setpoint patterns for three *n*-values

Setpoint #	*V*_tar_ (μV)	*%* of *I*_24_ *n* = 25	%of *I*_24_ *n* = 58	%of *I*_24_v*n* = 122
$\begin{array}{c} 1\\ 2\\ 3\\ 4\\ 5\\ 6 \end{array}]\;\text{Equal}\;\Delta I$		0.0	0.0	0.0
		11.7	14.3	15.5
		23.5	28.7	31.0
		35.2	43.0	46.5
		47.0	57.3	62.1
		58.7	71.7	77.6
$\begin{array}{c} 7\\ 8 \end{array}]\;\text{Transition region}$	0.004	70.5	86.0	93.1
	0.008	73.1	87.4	93.8
$\begin{array}{c} 9\\ 10\\ 11\\ 12\\ 13\\ 14\\ 15\\ 16\\ 17\\ 18\\ 19\\ 20\\ 21\\ 22\\ 23\\ 21 \end{array}]\;\text{Equal}\;\Delta \log(V)$	0.018	75.9	88.8	94.5
	0.032	77.3	89.5	94.9
	0.056	78.7	90.2	95.2
	0.100	80.2	90.9	95.6
	0.180	81.7	91.6	95.9
	0.320	83.2	92.4	96.3
	0.560	84.8	93.1	96.7
	1.000	86.3	93.8	97.0
	1.800	87.9	94.6	97.4
	3.200	89.5	95.3	97.8
	5.600	91.2	96.1	98.1
	10.00	92.9	96.9	98.5
	18.00	94.6	97.6	98.9
	32.00	96.4	98.4	99.3
	56.00	98.2	99.2	99.6
	100.0	100.0	100.0	100.0
25] Zero check		0.0	0.0	0.0

**Table 3:** Electric field windows used in computer aided data analysis

*E*_c_ criteria (μV/cm)	*E*_c_ top (μV/cm)	*E*_c_ bottom (μV/cm)	Ratio = top/bottom
0.1	1.5	0.06	25
0.2	2.4	0.12	20
0.5	3.6	0.20	18
1.0	5.0	0.30	17
2.0	8.0	0.60	13
5.0	15.0	1.50	10
10.0	21.0	3.00	7
20.0	50.0	10.0	5
50.0	100.0	20.0	5
100.0	140.0	28.0	5

**Table 4:** Coefficients of temperature variation for active simulators at various electric field criteria using V2, along with coefficients of variation for the critical currents determined using V2 and V3, and % difference in critical currents determined using V2 and V3

Simulator	Criteria (μV/cm)	*b* (A)	*m* (A/°C)	dev *I*_c_(V2) (%)	dev *I*_c_(V3) (%)	Δ*I*_c_ (%)
Active, 0.5 A	0.1	0.3680	−1.06 × 10^−5^	0.007	0.185	−0.080
50 mV, *n*=25	1.0	0.4047	−1.08 × 10^−5^	0.006	0.020	−0.001
40 points	10.0	0.4423	−1.19 × 10^−5^	0.005	0.006	0.000
23.6–27.9 °C	100.0	0.4944	−1.01 × 10^−5^	0.006	0.006	0.001
Active, 0.5 A	0.1	0.4354	−1.74 × 10^−5^	0.005	0.083	−0.052
50 mV, *n*=58	1.0	0.4535	−2.03 × 10^−5^	0.005	0.010	−0.004
40 points	10.0	0.4718	−1.99 × 10^−5^	0.005	0.005	−0.001
24.2–27.9 °C	100.0	0.4970	−2.47 × 10^−5^	0.008	0.008	0.000
Active, 0.5 A	0.1	0.4688	−3.35 × 10^−5^	0.005	0.040	−0.026
50 mV, *n*=122	1.0	0.4776	−2.68 × 10^−5^	0.003	0.005	−0.001
40 points	10.0	0.4869	−3.42 × 10^−5^	0.004	0.004	0.000
25.4–27.4 °C	100.0	0.4995	−3.41 × 10^−5^	0.007	0.007	0.000
Active, 50 A	0.1	43.582	−3.53 × 10^−3^	0.012	0.079	−0.009
50 mV, *n*=58	1.0	45.371	−3.42 × 10^−3^	0.005	0.010	0.002
120 points	10.0	47.203	−3.56 × 10^−3^	0.005	0.005	0.001
20.9–27.4 °C	100.0	49.726	−3.76 × 10^−3^	0.019	0.019	0.001
Active, 500 A	0.1	435.96	−4.57 × 10^−2^	0.008	0.085	−0.037
50 mV, *n*=58	1.0	453.76	−4.12 × 10^−2^	0.005	0.011	−0.002
40 points	10.0	472.01	−3.98 × 10^−2^	0.005	0.005	0.000
19.5–25.9 °C	100.0	496.99	−3.24 × 10^−2^	0.006	0.006	0.000
Active, 3000 A	0.1	2597.1	+ 9.12 × 10^−2^	0.031	0.081	−0.017
50 mV, *n* =58	1.0	2706.4	+5.77 × 10^−2^	0.023	0.025	0.000
40 points	10.0	2821.7	−8.98 × 10^−2^	0.017	0.017	0.000
20.2–26.1 °C	100.0	2978.9	−1.55 × 10^−1^	0.012	0.012	0.000
Active, 0.2 A	0.1	0.1749	+4.38 × 10^−6^	0.011	0.075	−0.030
20 mV, *n* =58	1.0	0.1822	+7.70 × 10^−7^	0.009	0.013	−0.005
40 points	10.0	0.1895	+7.25 × 10^−7^	0.011	0.010	−0.003
24.7–26.6 °C	100.0	0.1996	+4.10 × 10^−6^	0.016	0.016	−0.002
Active, 0.3 A	0.1	0.2612	+9.93 × 10^−6^	0.011	0.069	−0.013
30 mV, *n*=58	1.0	0.2721	+6.51 × 10^−6^	0.009	0.012	−0.004
40 points	10.0	0,7«31	+1.66 × 10^−6^	0.008	0.008	−0.001
24.4–28.8 °C	100.0	0.2988	−1.91 × 10^−5^	0.012	0.013	0.000

**Table 5:** Coefficients of temperature variation for the hybrid and passive simulators at various electric field criteria using V2, along with coefficients of variation for the critical currents determined using V2 and V3, and *%* difference in critical currents determined using V2 and V3

Simulator	Criteria (μV/cm)	*b* (A)	*m* (A/°C)	dev *I*_c_(V2) (%)	dev *I*_c_(V3) (%)	Δ*I*_c_ (%)
Hybrid, 2 A	0.1	1.6200	+1.30 × 10^−5^	0.008	0.162	0.225
120 points	1.0	1.8006	+1.12 × 10^−5^	0.007	0.020	0.027
19.6–26 °C	10.0	1.9864	+1.10 × 10^−5^	0.005	0.006	0.003
	100.0	2.2150	+2.39 × 10^−5^	0.012	0.012	0.000
Passive, 2 A	0.1	1.8476	−6.18 × 10^−3^	0.024	0.262	−0.076
120 points	1.0	2.0047	−5.56 × 10^−3^	0.013	0.030	−0.011
14.2–29.0 °C	10.0	2.1680	−4.96 × 10^−3^	0.009	0.010	−0.005
	100.0	2.3804	−4.46 × 10^−3^	0.013	0.013	−0.004
Passive, 25 A	0.1	20.982	−6.67 × 10^−2^	0.013	0.182	−0.097
60 points	1.0	23.042	−6.38 × 10^−2^	0.012	0.021	−0.008
13.8–24.2 °C	10.0	25.017	−5.74 × 10^−2^	0.008	0.008	−0.001
	100.0	27.473	−5.14 × 10^−2^	0.009	0.009	−0.002
Passive, 50 A	0.1	41.098	−1.31 × 10^−1^	0.017	0,186	−0.110
80 points	1.0	45.135	−1.26 × 10^−1^	0.011	0.020	−0.007
14.3–24 °C	10.0	48.997	−1.13 × 10^−1^	0.007	0.007	−0.002
	100.0	53,805	−1.01 × 10^−1^	0.014	0.014	−0.003

**Table 6:** 50 A passive simulator temperature coefficients at low electric field criteria using V2, along with coefficients of variation for the critical currents determined using V2 and V3, and % difference in critical currents determined using V2 (digital nanovoltmeter) and V3 (analog nanovoltmeter)

Simulator	Criteria (μV/cm)	*b* (A)	*m* (A/°C)	dev *I*_c_ (V2) (%)	dev *I*_c_ (V3) (%)	Δ*I*_c_ (%)
Passive, 50 A	0.01	36.775	−1.41×10^−1^	0.051	0.749	0.099
40 points	0.10	41.154	−1.36×10^−1^	0.019	0.089	0.010
18.9–28.3 °C	1.00	45.146	−1.25×10^−1^	0.009	0.010	−0.001

**Table 7:** 50 A passive simulator temperature coefficients at low electric field criteria using V2, along with coefficients of variation for the critical currents determined using V2 and V3, and % difference in critical currents determined using V2 (digital nanovoltmeter) and V3 (digital nanovoltmeter)

Simulator	Criteria (μV/cm)	*b* (A)	*m* (A/°C)	dev *I*_c_ (V2) (%)	dev *I*_c_ (V3) (%)	Δ*I*_c_ (%)
Passive, 50 A	0.01	36.819	−1.43×10^−1^	0.051	1.99	−0.082
120 points	0.10	41.150	−1.36×10^−1^	0.023	0.288	−0.128
16.2–26.2 °C	1.00	45.141	−1.25×10^−1^	0.010	0.024	−0.004

**Table 8:** Stability of critical currents determined using R2 over time for the hybrid, passive, and active simulators. Δ*I*_c_ calculated by 100% [*I*_c_(day #x)–*I*_c_(day #l)]/*I*_c_(day *#*1)

Simulator	Criteria (μV/cm)	Δ*I*_c_=100% [*I*_c_(day #x)–*I*_c_(day #l)]/*I*_c_(day #1) (# in parentheses indicates day #x)		
Hybrid	0.1	0.048 (7)	−0.042 (8)	−0.002 (56)
2A	1.0	0.010 (7)	−0.019 (8)	−0.007 (56)
	10.0			−0.007 (56)
	100.0			−0.008 (56)
Passive	0.1	−0.001 (2)	0.025 (8)	0.042 (106)
2A	1.0	0.011 (2)	0.009 (8)	0.012 (106)
	10.0	0.000 (2)	0.000 (8)	0.000 (106)
	100.0	−0.002 (2)	0.002 (8)	−0.034 (106)
Passive	0.1	−0.027 (8)	−0.036 (9)	−0.029 (139)
50 A	1.0	0.006 (8)	0.000 (9)	0.002 (139)
	10.0			0.001 (139)
	100.0			−0.035 (139)
Active	0.1	0.000 (6)	−0.062 (69)	−0.042 (104)
0.5 A	1.0	−0.010 (6)	−0.043 (69)	−0.055 (104)
	10.0	−0.016 (6)	−0.041 (69)	−0.055 (104)
	100.0	−0.008 (6)	−0.028 (69)	−0.043 (104)

**Table 9:** Comparison of the equivalent shunt voltage for an active simulator at a temperature of 26 °C and *n* =58 using different power supplies. *E*_c_= 10 μV/cm, data taken on R2, 50 mV shunt resistor rating

Day #	Power supply rating, A	Input *R* (mΩ)	*I*_c_(*T* = 26°C) (A)	Shunt *V* (mV)	% difference w.r.t #135
135	10 A	100.	0.4712	47.124	0.000
140	10 A	100.	0.4712	47.116	−0.017
178	1000 A	1.	47.111	47.111	−0.027
195	1000 A	0.1000	470.97	47.097	−0.057
196	3000 A	0.0167	2819.3	46.989	−0.286
198	3000 A with filtering	0.0167	2823.3	47.055	−0.146
203	10 A	100.	0.4710	47.105	−0.040
238	10 A	100.	0.4710	47.098	−0.055

## Footnotes

1 Certain commercial equipment, instruments, or materials mentioned in this paper might be indirectly identified by their particular properties. Such identification does not imply recommendation or endorsement by the National Institute of Standards and Technology, nor does it imply that the materials or equipment identified are necessarily the best available for the purpose.
